# Surface growth of *Pseudomonas aeruginosa* reveals a regulatory effect of 3-oxo-C_12_-homoserine lactone in the absence of its cognate receptor, LasR

**DOI:** 10.1128/mbio.00922-23

**Published:** 2023-09-21

**Authors:** Thays de Oliveira Pereira, Marie-Christine Groleau, Eric Déziel

**Affiliations:** 1 Centre Armand-Frappier Santé Biotechnologie, Institut National de la Recherche Scientifique (INRS), Laval, Québec, Canada; Emory University School of Medicine, Atlanta, Georgia, USA

**Keywords:** quorum sensing, surface sensing, virulence, microbial communities, biofilms

## Abstract

Successful colonization of a multitude of ecological niches by the bacterium *Pseudomonas aeruginosa* relies on its ability to respond to concentrations of self-produced signal molecules. This intercellular communication system known as quorum sensing (QS) tightly regulates the expression of virulence determinants and a diversity of survival functions, including those required for social behaviors. In planktonic cultures of *P. aeruginosa*, the transcriptional regulator LasR is generally considered on top of the QS circuitry hierarchy; its activation relies on binding to 3-oxo-C_12_-homoserine lactone (3-oxo-C_12_-HSL), a product of LasI synthase. Transcription of *lasI* is activated by LasR, resulting in a positive feedback loop. Few studies have looked at the function of QS during surface growth even though *P. aeruginosa* typically lives in biofilm-like communities under natural conditions. Here, we show that surface-grown *P. aeruginosa*, including prototypical strain PA14, produces 3-oxo-C_12_-HSL in the absence of LasR. This phenotype is commonly observed upon surface association in naturally occurring environmental and clinical LasR-defective isolates, suggesting a conserved alternative function for the signal. Notably, in surface-grown cultures, 3-oxo-C12-HSL reaches higher levels than planktonic cells, and there is a delayed timing of its production. Accordingly, 3-oxo-C_12_-HSL upregulates the autologous expression of pyocyanin and LasR-controlled virulence determinants in neighboring cells even in the absence of the cognate regulator LasR. This highlights a possible role for 3-oxo-C_12_-HSL in shaping community responses and provides a possible evolutive benefit for mixed populations to carry LasR-defective cells, a common feature of natural populations of *P. aeruginosa*.

**IMPORTANCE:**

The bacterium *Pseudomonas aeruginosa* colonizes and thrives in many environments, in which it is typically found in surface-associated polymicrobial communities known as biofilms. Adaptation to this social behavior is aided by quorum sensing (QS), an intercellular communication system pivotal in the expression of social traits. Regardless of its importance in QS regulation, the loss of function of the master regulator LasR is now considered a conserved adaptation of *P. aeruginosa*, irrespective of the origin of the strains. By investigating the QS circuitry in surface-grown cells, we found an accumulation of QS signal 3-oxo-C_12_-HSL in the absence of its cognate receptor and activator, LasR. The current understanding of the QS circuit, mostly based on planktonic growing cells, is challenged by investigating the QS circuitry of surface-grown cells. This provides a new perspective on the beneficial aspects that underline the frequency of LasR-deficient isolates.

## INTRODUCTION

Bacteria are social organisms that often respond to environmental cues in coordination. *Pseudomonas aeruginosa* is a highly adaptable Gram-negative bacterium that colonizes diverse ecological niches. The flexibility of this opportunist human pathogen is aided by several regulatory networks, assuring proper responses to changing environmental conditions. Quorum sensing (QS) is a gene expression regulation mechanism based on the production, release, detection and response to diffusible signaling molecules that synchronize the transcription of target genes in a population density-dependent manner (1). In *P. aeruginosa*, three interlinked QS systems regulate the expression of hundreds of genes—including several encoding virulence determinants (2). In this bacterium, QS regulation is structured as a hierarchical network composed of two *N*-acyl homoserine lactone (AHL)-based circuits (*las* and *rhl*) and the *pqs* system, which relies on signaling molecules of the 4-hydroxy-2-alkylquinoline (HAQ) family. The *las* and *rhl* systems comprise an AHL synthase (LasI and RhlI) responsible for the syntheses of *N*‐(3‐oxododecanoyl)‐L‐homoserine lactone (3-oxo‐C_12_‐HSL) and *N*‐butanoyl‐L‐homoserine lactone (C_4_‐HSL), respectively (3, 4). These autoinducers activate their cognate LuxR-type transcriptional regulators—LasR and RhlR, which, in turn, can induce the transcription of target QS-regulated genes. Under standard laboratory conditions, the *las* system is generally considered to be atop the regulatory hierarchy. Once activated by the binding with its cognate autoinducer, LasR regulates several virulence traits such as elastase LasB (*lasB*) (5, 6). LasR also induces the transcription of LasI synthase coding gene, creating a positive feedback loop (7). The *pqs* system relies on the LysR-type transcriptional regulator MvfR (also known as PqsR) (8, 9). The latter directly activates the operons *pqsABCDE* and *phnAB*, both of which are required for HAQ biosynthesis and indirectly regulates the expression of many other QS-regulated genes via PqsE (8, 10 – 14). MvfR has dual ligands as it can be induced by 4‐hydroxy‐2‐heptylquinoline (HHQ) and the *Pseudomonas* quinolone signal (PQS; 3,4-dihydroxy-2-alkylquinoline), both of which are members of the HAQ family (15, 16). The *rhl* and *pqs* circuits are directly and positively regulated by LasR, which induces the transcription of *rhlR* and *rhlI* as well as *mvfR* (13, 15, 17, 18).

In addition to sensing the surrounding chemical environment, bacteria are also responsive to mechanical signals, such as those involved in the physical encounter of the cell with surfaces or with each other. Indeed, several behaviors are specific to life on surfaces, including movement on semi-solid (swarming motility) and solid surfaces (twitching motility) as well as biofilm formation (19 – 21). Not surprisingly, virulence is also induced by surface attachment as many infection strategies require contact with the host (22 – 24). Even though QS and surface sensing regulate many of the same social behaviors, little is known about how these different regulatory cues converge to modulate bacterial responses. Exploring the link between surface sensing and QS is particularly relevant as *P. aeruginosa* readily adopts a surface-attached mode of growth as biofilms in its natural habitats. Biofilms are organized communities encased in a self-produced exopolymeric matrix. In the context of infections, biofilms contribute to host immune evasion and delay antibiotic penetration (25, 26). In fact, *P. aeruginosa* persists as biofilms in the lungs of people with cystic fibrosis, a genetic disease (27).

While the emergence of LasR-defective mutants has long been associated with adaptation to the CF lung environment (28 – 31), it is actually a common feature of *P. aeruginosa* from diverse environments (32, 33). Interestingly, some LasR-defective isolates, known as RhlR active independently of LasR (RAIL), retain a functional RhlR regulator (31, 32, 34 – 37). Their sustained QS responses are in line with our previous report showing that in the presence of a non-functional LasR, RhlR acts as a surrogate activator for a set of LasR-regulated genes (38). It is noteworthy that in the wild-type *P. aeruginosa* strain PA14 background, surface sensing upregulates *lasR* and that surface-grown cells induce LasR targets more strongly than their planktonic counterpart (39). Thus, surface sensing appears to sensitize cells to the cognate autoinducer 3-oxo‐C_12_‐HSL. Considering the prevalence of LasR-defective mutants, which neither produce nor respond to 3-oxo‐C_12_‐HSL, we wondered how *P. aeruginosa* would respond to surface attachment as biofilm formation is essential to bacterial physiology and pathology.

In this study, we investigated the effect of surface sensing on QS responses of LasR-defective strains. We found that upon surface attachment, LasR becomes dispensable to the production of 3-oxo‐C_12_‐HSL. This response is conserved among naturally occurring environmental and clinical LasR-defective isolates. Production of 3-oxo‐C_12_‐HSL modulates the production of virulence factors at individual (LasR-defective background) and community levels (mixed with LasR-responsive cells). We propose that the production of 3-oxo‐C_12_‐HSL by LasR-negative cells, modulating biological bacterial responses on diverse levels, has a positive role in shaping community responses of the population.

## MATERIALS AND METHODS

### Bacterial strains and growth conditions

Bacterial strains and plasmids used in this study are listed in Tables 1 and 2, respectively. Oligonucleotides used are listed in Table S1. Bacteria were routinely grown in tryptic soy broth (TSB; BD Difco, Canada) at 37°C in a TC-7 roller drum (NB, Canada) at 240 rpm or on lysogeny broth (LB; BD Difco, Canada) agar plates. For quantification of QS signaling molecules and related data, King’s A broth (planktonic growth) or King’s A agar (surface-associated growth) supplemented with 100 µM FeCl_3_ was used (40). For the latter, sterile King’s A agar was poured into each well of a 96-well plate (200 µL per well) and allowed to solidify at the center of a biosafety cabinet. When needed, the following concentrations of antibiotics were included: for *Escherichia coli,* 100 µg/mL carbenicillin, 15 µg/mL gentamicin, and 15 µg/mL tetracycline were used; diaminopimelic acid (DAP) was added to cultures of the auxotroph *E. coli* χ7213 at 62.5  µg/mL; Irgasan (20 µg/mL) was used as a counter-selection agent against *E. coli*; and for *P. aeruginosa*, 300 µg/mL carbenicillin, 30 µg/mL gentamicin, and tetracycline were added at 125 µg/mL (solid) or 75 µg/mL (liquid).

**TABLE 1:** Strains used in this study

Strain	Lab ID #	Relevant genotype or description	Reference
*P. aeruginosa*			
PA14	ED14	Clinical isolate from a human burn patient UCBPP-PA14	(41)
PA14 ∆*lasR*	ED4409	PA14 derivate; unmarked in-frame *lasR* deletion	This study
PA14 ∆*lasI*	ED4539	PA14 derivate; unmarked in-frame *lasI* deletion	(42)
PA14 ∆*rhlR*	ED4406	PA14 derivate; unmarked in-frame *rhlR* deletion	This study
PA14 *lasR^-^ rhlR^-^*	ED266	PA14 derivate; marked deletion of *lasR* (*lasR*::Gm) and *rhlR* (*rhlR*::Tc)	(38)
PA14 ∆*lasR* ∆*rhlI*	ED4541	PA14 derivate; unmarked in-frame double *lasR* and *rhlI* deletion	This study
PA14 *lasR^-^* ∆*pqsE*	ED247	PA14 derivate; marked deletion of *lasR* (*lasR*::Gm) and an unmarked *pqsE* deletion	(13)
PA14 ∆*lasR* ∆*lasI*	ED4540	PA14 derivate; unmarked in-frame double *lasR* and *lasI* deletion	This study
PA14 ∆*lasR*∆*rhlR*	ED4545	PA14 derivate; unmarked in-frame double *lasR* and *rhlR* deletions	This study
PA14 ∆*lasR* ∆*lasI* ∆*rhlI attB*::CTX *phzA1-lux*	ED4544	PA14 derivate; unmarked in-frame triple *lasR, lasI,* and *rhlI* deletion carrying the chromosomal *phzA1-lux* reporter	This study
PA14 ∆*lasR* ∆*lasI attB*::CTX *phzA1-lux*	ED4591	PA14 derivate; ED4540 carrying the chromosomal *phzA1-lux* reporter	This study
PA14 ∆*lasI attB*::CTX *lasB-lux*	ED4543	PA14 derivate; ED4539 carrying the chromosomal *lasB-lux* reporter	This study
PA14 ∆*lasR attB*::CTX *lasI-lux*	ED4542	PA14 derivate; ED4409 carrying the chromosomal *lasI-lux* reporter	This study
PA14 ∆*lasR* ∆*pilT*	ED4556	PA14 derivate; unmarked in-frame double *lasR* and *pilT* deletion	This study
PA14 ∆*lasR pilU*	ED4557	PA14 derivate; unmarked in-frame *lasR* and marked *pilU* mutant (*pilU::*MrT7)	This study
18G	ED4592	Oil-contaminated soil isolate	(43)
32R	ED4593	Oil-contaminated soil isolate	(43)
78RV	ED4590	Oil-contaminated soil isolate	(43)
E41	ED4160	Cystic fibrosis isolate	(31)
E113	ED4144	Cystic fibrosis isolate	(31)
E167	ED4152	Cystic fibrosis isolate	(31)
E113 ∆*rhlR*	ED4145	E113 derivate carrying an unmarked deletion in the *rhlR* gene	(37)
E167 ∆*rhlR*	ED4153	E167 derivate carrying an unmarked deletion in the *rhlR* gene	(37)
*E. coli*			
SM10(λ*pir*)	ED222	*thi thr leu tonA lacY supE recA*::*RP4-2*-Tc::*Mu* Km λ*pir*	Lab collection
χ7213	ED743	*thr-1 leuB6 fhuA21 lacY1 glnV44 recA1* Δ*asdA4* Δ(*zhf-2*::Tn*10*) *thi-1 RP4-2-*Tc::*Mu*[λ *pir*]	Lab collection

**TABLE 2:** Plasmids used in this study

Plasmid	Description	Reference or source
pTOP01	pEX18Ap∆*lasR*; gene replacement vector for the in-frame deletion of *lasR* by allelic recombination, Carb^r^	This study
pTOP02	pEX18Ap∆*rhlR;* gene replacement vector for the in-frame deletion of *rhlR* by allelic recombination, Carb^r^	This study
pTOP03	pEX18Ap∆*rhlI*; gene replacement vector for the in-frame deletion of *rhlI* by allelic recombination, Carb^r^	This study
pTOP04	pEX18Ap∆*pilT*; gene replacement vector for the in-frame deletion of *pilT* by allelic recombination, Carb^r^	This study
pEX18Gm∆*lasI*	Gene replacement vector for the in-frame deletion of *lasI* by allelic recombination, Gm^r^	(42)
pTOP05	Promoter of *lasI* in mini-CTX-*lux*, Tet^r^	This study
pCDS101	Promoter of *phz1* in mini-CTX-*lux*, Tet^r^	(44)
pCTX-1-P*_lasB_-lux*	Promoter of *lasB* in mini-CTX-*lux*, Tet^r^	(45)
pME3853	*lasI′–′lacZ* translational fusion, Tet^r^	(46)

### Construction of in-frame deletion mutants

An allelic exchange technique based on the use of a suicide vector was used to construct gene knockout deletions (47). Mutant alleles, flanked by regions of homology to the recipient chromosome, were synthesized *in vitro* by PCR from PA14 genomic DNA and then cloned into the allelic exchange vector pEX18Ap (yielding pTOP01, pTOP02, pTOP03, and pTOP04). Plasmids were assembled from purified PCR products and a restriction enzyme-cleaved plasmid backbone by employing a seamless strategy of ligation-independent cloning (pEASY -Uni Seamless Cloning and Assembly Kit, TransGen Biotech Co.). These suicide vectors were transferred into *P. aeruginosa* by conjugation with an *E. coli* donor strain (SM10). Carbenicillin was used to select recipient merodiploid cells, and *E. coli* donor cells were counter-selected using Irgasan. Double-crossover mutants were isolated by sucrose counter-selection and confirmed by PCR.

### Inactivation of *pilU* gene

Transfer of transposon insertion (::MrT*7*) from the PA14 non-redundant transposon insertion mutant library was used (48) to inactivate *pilU*. Genomic DNA from *pilU*::MrT*7* (mutant ID # 53607) was extracted and transformed into the recipient PA14 ∆*lasR* background. Gentamicin (15 µg/mL) was used to select transformants.

### Construction of reporter strains

The promoter region of *lasI* was PCR amplified from PA14 genomic DNA. pTOP05 (mini-CTX-*lasI-lux*) was constructed by the assembly of the purified PCR product and the enzyme-cleaved mini-CTX-lux backbone (49). pTOP05, pCTX-1-P*_lasB_-lux,* and pCDS101 were integrated into the *attB* chromosomal site of PA14 and isogenic mutants by conjugation on LB agar plates. Selection was performed on LB agar plates containing tetracycline. The non-integrative plasmid pME3853 carrying a *lasI′–′lacZ* translational fusion was transformed into electrocompetent *P. aeruginosa* cells and selected with tetracycline (50).

### Gene expression reporter measurements

For *lux* reporter readings, luminescence was measured using a Cytation3 multimode plate reader (BioTek Instruments, USA). Relative light units (RLUs) were normalized by colony-forming units per milliliter (reported in RLU CFU^−1^). The activity of *lacZ* reporters was determined by β-galactosidase activity and was normalized by CFU (reported in Miller units per cell) (51). When mentioned, AHLs were added to a final concentration of 1.5 µM of C_4_-HSL and 3 µM of 3-oxo-C_12_-HSL from stocks prepared in high-performance liquid chromatography (HPLC)-grade acetonitrile. Acetonitrile only was added in controls.

### Quantification of QS signaling molecules

Concentration of 3-oxo-C_12_-HSL was measured for bacteria grown in liquid King’s A (planktonic growth) and on King’s A agar (surface growth) using HPLC/tandem mass spectrometry (LC/MS/MS), with modifications of the previously described protocol (52). Quantification was performed at indicated times post-inoculation in both growth conditions. For planktonic growth, overnight cultures grown on TSB were diluted to OD_600_ of 0.1 in fresh King’s A medium. At the given time-points, cultures were mixed with acetonitrile containing the internal standard tetradeuterated 4-hydroxy-2-heptylquinoline (HHQ-d_4_), in a 4:1 ratio of culture to solvent (HHQ-d_4_ final concentration of 3 ppm). Bacterial suspension was vortexed and centrifuged at maximum speed for 10 min in order to pellet bacterial cells. The resulting mixture was transferred into vials for LC/MS/MS analyses. Alternatively, for cells grown on agar surfaces, overnight cultures on TSB were diluted to OD_600_ of 0.05 in TSB medium. Cultures were grown until an OD_600_ of 1 and agar plugs were inoculated with 5 µl of bacterial suspension. Plates were incubated at 37°C and samples were collected at the indicated time-points. Each sample was composed of two agar plugs mixed with 1 mL of acetonitrile containing the internal standard. This mixture was incubated at 4°C for 16h under gentle agitation, optimizing the diffusion of signaling molecules from the agar to the solvent. After incubation, the mixture was centrifuged at maximum speed for 10 min and the resulting supernatant was transferred into a LC/MS vial. All samples were injected using an HPLC Waters 2795 (Mississauga, ON, Canada) on a Kinetex C8 column (Phenomenex) with an acetonitrile-water gradient containing 1% acetic acid. The detector was a tandem quadrupole mass spectrometer (Quattro premier XE; Waters) equipped with a Z-spray interface using electrospray ionization in positive mode (ESI+). Nitrogen was used as a nebulizing and drying gas at flow rates of 15 and 100 ml · min^−1^, respectively. Concentration was normalized by CFUs per mL^-1^ and reported in ng CFU^-1^. All experiments were performed in triplicates and repeated at least twice independently

### Pyocyanin quantification

Quantification of pyocyanin produced by surface-grown cells was performed similarly to that described in a previous study (53). Overnight cultures were diluted and grown in TSB until an OD_600_ of 1. At this point, 5 µL was used to inoculate agar plugs from a 96-well plate containing King’s A agar supplemented with FeCl_3_ (200 µL per well). Plates were incubated at 37°C for 24 h. Pyocyanin was extracted in 500 µL of chloroform from two agar plugs (by replicate). Tubes were vortexed and centrifuged for 3 min at 12,000 × *g*. Then, 200 µL of the organic phase was recovered in a new tube, and a second chloroform extraction was performed on the plugs. The organic phase (400 µL) was acidified with 500 µL 0.2 N HCl and vortexed. The samples were centrifuged for 3 min at 12,000 × *g,* and the absorbance of the pink aqueous phase was read at OD_520 nm_. Blank was performed by pyocyanin extraction from uninoculated agar plugs. Values were corrected by colony-forming units per milliliter from samples prepared in the same conditions.

### *Drosophila melanogaster* feeding assay

Fruit flies (*D. melanogaster*) were infected orally in a feeding assay model (54). Male flies (4 to 6 days old) were anesthetized under a gentle stream of carbon dioxide and separated into vials, each containing 10 males. Each strain (or condition) tested was composed of three independent vials, totalizing 30 flies. Vials were prepared with 5 mL of a solution of sucrose agar (5% of sucrose and 1.5% agar). Once solidified, a sterile filter disk was placed on the surface. Prior to infection, bacteria were grown in 6 mL of TSB until an OD_600_ of 3. At this point, the bacterial suspension was centrifuged 3 min at 12,000 × *g,* and the pellet was resuspended in 100 µL of sterile 5% sucrose and dispensed on the filter papers. Sterile 5% sucrose alone was used as control. Males were starved 6–8 h prior to the infection. Flies were kept at 25°C and about 50% humidity. They were subjected to 12-h light/dark cycles. Mortality was monitored daily for 8 days. The experiment was performed twice, each time in triplicate.

## RESULTS

### Surface growth induces production of 3-oxo-C_12_-HSL in the absence of LasR

**Fig 1:**
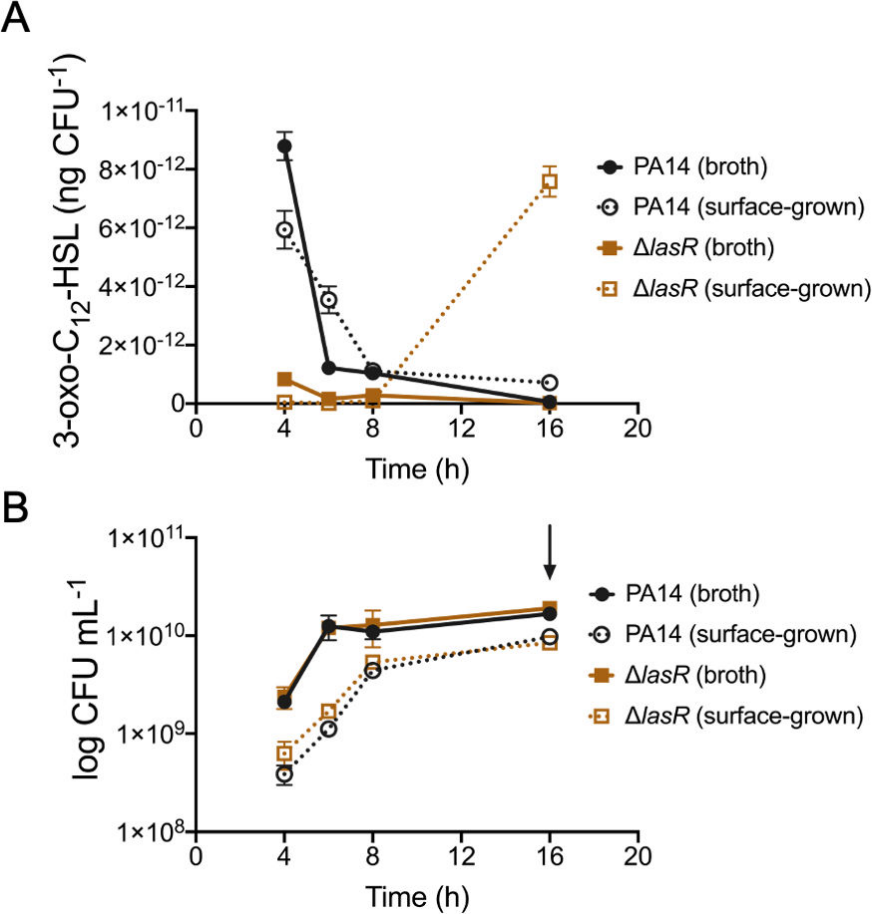
Surface growth induces 3-oxo-C_12_-HSL production in PA14 LasR-null strain. (**A**) 3-oxo-C_12_-HSL concentration was measured in PA14 and the isogenic Δ*lasR* mutant (PA14 Δ*lasR*) at different time points during planktonic (broth culture) and surface growth (surface of agar-solidified culture media) by liquid chromatography/mass spectrometry. Values were normalized by the viable cell counts and shown in nanograms per CFU (**B**) Growth in broth and surface conditions was determined by the count of viable cells per milliliter (CFU mL^−1^). The arrow indicates the time point at which 3-oxo-C_12_-HSL is induced in a Δ*lasR* mutant in (**A**). The values are means ± standard deviation (error bars) from three replicates.

In *P. aeruginosa* prototypical strains such as PA14**,** the quorum-sensing regulatory cascade is considered to be primarily activated by the *las* system. LasR, once activated by the binding of 3-oxo-C_12_-HSL, regulates the transcription of target genes, including the gene coding LasI synthase. This process induces the production of more 3-oxo-C_12_-HSL, resulting in a positive feedback loop. In standard laboratory liquid cultures of *P. aeruginosa*, production of 3-oxo-C_12_-HSL peaks early and decreases overtime [(11); Fig. 1A]. We note the same pattern of production in wild-type *P. aeruginosa* PA14 (WT) cells grown on an agar surface (Fig. 1A). Surprisingly, in a LasR-negative background, the production pattern of 3-oxo-C_12_-HSL is influenced by suspended vs surface culture conditions (Fig. 1). As expected, production of the LasR ligand is barely detectable at the stationary phase of a Δ*lasR* mutant in broth cultures. However, its concentration is elevated during surface growth (Fig. 1A and B). In WT culture, the peak concentration is observed during the exponential growth phase, while it shifts to late-stationary phase in the Δ*lasR* mutant, solely when growing on the surface. This shift might indicate a role for other regulators in the activation of *lasI* transcription in the absence of LasR.

### Production of 3-oxo-C_12_-HSL and expression of *lasI* are RhlR dependent in LasR-negative backgrounds

**Fig 2:**
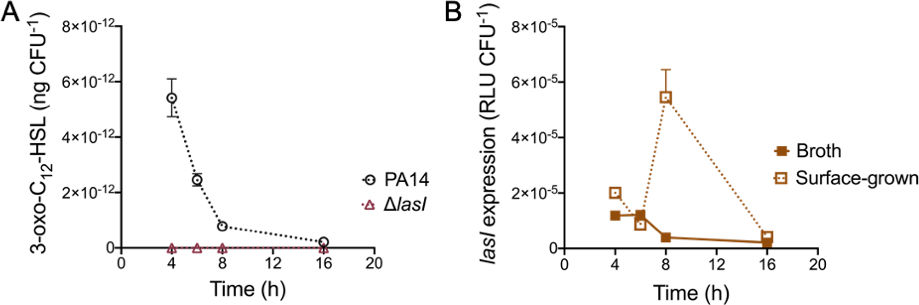
Transcription of *lasI* can occur in the absence of LasR in cells growing on a surface. (**A**) 3-oxo-C_12_-HSL was measured in PA14 and its isogenic Δ*lasI* mutant at different time points during surface growth by liquid chromatography/mass spectrometry. (**B**) Transcription activity from the chromosomal *lasI-lux* reporter in a Δ*lasR* background.

Expression of the gene coding the LasI synthase, responsible for the synthesis of 3-oxo-C_12_-HSL, is typically considered to be regulated by LasR. Therefore, little to no production of this AHL is expected in LasR-defective strains, which is what is observed in planktonic cultures. However, upon surface growth, 3-oxo-C_12_-HSL is produced in the absence of LasR. To make sure the production of 3-oxo-C_12_-HSL in this condition still requires LasI activity, we measured concentrations of this AHL in a Δ*lasI* mutant grown under the same surface-associated conditions. As expected, 3-oxo-C_12_-HSL is not detectable in a Δ*lasI* mutant, irrespective of the growth phase (Fig. 2A). The concentrations of this signal molecule were also assessed in the Δ*lasR*Δ*lasI* double mutant, and similarly to the Δ*lasI* mutant, we detected no 3-oxo-C_12_-HSL (data not shown). These results suggest that transcription of *lasI* can occur in the absence of LasR upon surface growth. To further investigate this, we measured the activity of a chromosomal *lasI-lux* reporter in a Δ*lasR* background in both planktonic and surface-grown cells. In agreement with the production of 3-oxo-C_12_-HSL, transcription of *lasI* was observed in LasR-negative background grown on a surface (Fig. 2B).

**Fig 3:**
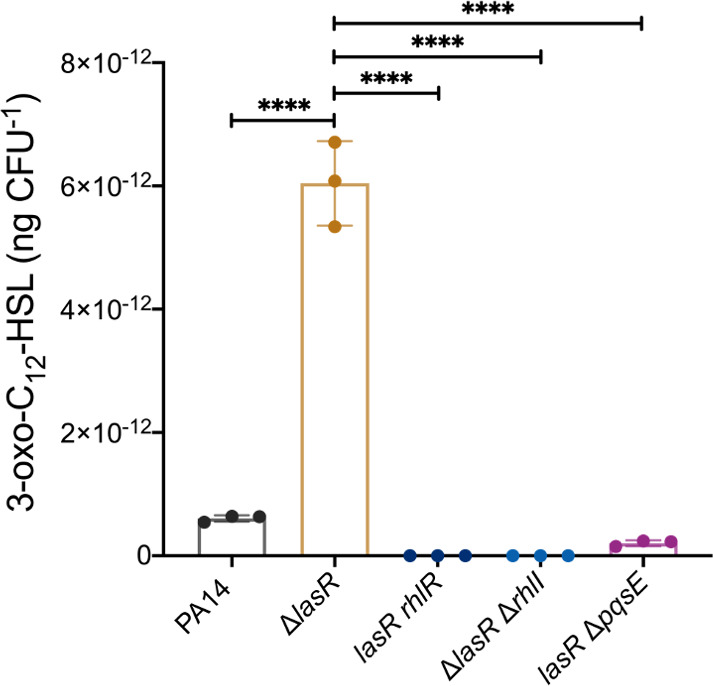
Activity of the Rhl system is required to induce the production of 3-oxo-C_12_-HSL upon surface growth. 3-oxo-C_12_-HSL was measured in PA14, isogenic single-mutants Δ*lasR* and Δ*rhlR*, and the double-mutants *lasR-rhlR-*, Δ*lasR*Δ*rhlI*, and *lasR-*Δ*pqsE* at 16 h of surface growth by LC/MS. Concentration was normalized by the viable cell count. The values are means ± standard deviation (error bars) from three replicates. One-way analysis of variance (ANOVA) and Tukey’s multiple comparisons posttest were used to quantify statistical significance. ****P ≤ 0.0001.

We have previously reported indications that RhlR can act as a surrogate regulator of LasR-dependent factors in the absence of LasR (38). In *P. aeruginosa* planktonic cultures, this activation is seen by the production of 3-oxo-C_12_-HSL at late stationary phase in LasR-negative backgrounds. However, as shown in Fig. 1A, the concentration of this AHL in a Δ*lasR* mutant in broth cultures remains extremely low early on. In contrast, surface growth readily induces production and the corresponding upregulation of *lasI* transcription in a Δ*lasR* mutant (Fig. 1A and 2B). To verify if RhlR is responsible for this upregulation, we measured concentrations of 3-oxo-C_12_-HSL in a Δ*rhlR* and a double *lasR rhlR* mutant (Fig. 3; Fig. S1) upon surface growth. The production profile of 3-oxo-C_12_-HSL is similar between the WT and a Δ*rhlR* mutant, peaking at exponential growth phase and decaying overtime (Fig. S1). On the other hand, the concomitant inactivation of *lasR* and *rhlR* abrogates 3-oxo-C_12_-HSL production, which concurs with our previous finding of RhlR being an alternative activator of LasI in LasR-negative backgrounds (Fig. 3; Fig. S1). This result suggested that RhlR in surface-grown cells mediates the transcription of *lasI*. To verify the potential transcriptional regulatory activity of RhlR on the expression of *lasI*, we introduced the transcriptional *lasI-lux* reporter construct in a double *lasR rhlR* mutant. Surprisingly, the transcriptional profile of *lasI* in surface-grown cells of this double mutant is similar to the 3-oxo-C_12_-HSL-producing Δ*lasR* mutant (Fig. S2A), despite the absence of production of this signal. To further establish a connection between the RhlR-dependent production of 3-oxo-C_12_-HSL and *lasI* expression during surface growth, we assessed the translation of *lasI* using a reporter fusion in the double Δ*lasR*Δ*rhlR* mutant and Δ*lasR*. Similar to the transcription findings for *lasI* (Fig. 2B), the translation also correlates with the production of 3-oxo-C_12_-HSL in the Δ*lasR* mutant (Fig. S2B). However, in the absence of both LasR and RhlR, no translation of *lasI* is detected, thus explaining the lack of 3-oxo-C_12_-HSL production by the Δ*lasR*Δ*rhlR* mutant (Fig. S2B).

Thus, in the absence of LasR, surface-grown cells rely on the activity of the *rhl* system to control QS-regulated factors, including the production of 3-oxo-C_12_-HSL. Since the full activity of RhlR depends on both C_4_-HSL and PqsE (13), we measured the concentration of 3-oxo-C_12_-HSL in the double mutants Δ*lasR*Δ*rhlI* and *lasR* Δ*pqsE* in order to further elucidate the role of the Rhl system in this mechanism. As expected, inactivating *rhlI* or *pqsE* in a *lasR* background severely affects the production of 3-oxo-C_12_-HSL (Fig. 3) and confirms that the production of 3-oxo-C_12_-HSL by LasR cells growing on a surface is dependent on the RhlR-mediated regulation of *lasI*.

### Induction of the production of 3-oxo-C_12_-HSL upon surface growth is a widespread response among *P. aeruginosa* strains

**Fig 4:**
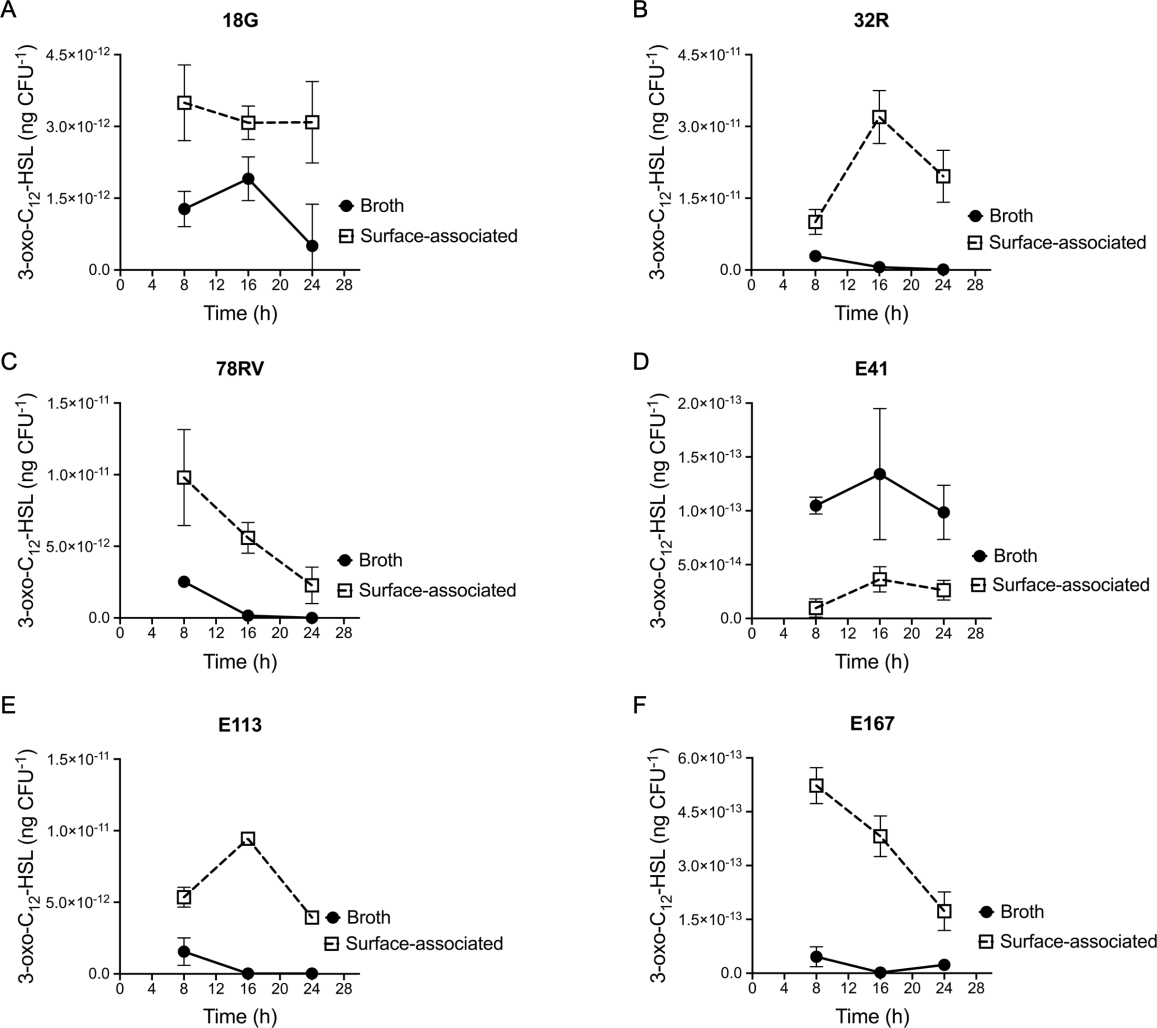
Production of 3-oxo-C_12_-HSL is a widespread feature among LasR-defective strains growing on a surface. 3-oxo-C_12_-HSL was measured at different time points during planktonic and surface growth by LC/MS of naturally evolved LasR-defective strains. (**A**) 18G. (**B**) 32R. (**C**) 78RV. (**D**) E41. (**E**) E113. (**F**) E167. Concentration was normalized by viable cell count and is given in nanograms per CFU. The values are means ± standard deviation (error bars) from three replicates.

Conserved regulation pathways strongly suggest the importance of bacterial responses to their fitness (55). We have observed that surface growth induces production of 3-oxo-C_12_-HSL in an engineered *lasR* deletion mutant of *P. aeruginosa* PA14. To verify if this response is restricted to this prototypical strain, we measured concentrations of this AHL in six naturally occurring LasR-defective *P. aeruginosa* isolates: three strains we recently identified among a collection of environmental isolates (32), and the other three are LasR-defective CF clinical isolates (E41, E113, and E167) from the Early *Pseudomonas* Infection Control (EPIC) study (31, 37). The timing of sampling was chosen based on the 3-oxo-C_12_-HSL production profile of PA14 Δ*lasR*, which peaks at the late exponential phase (Fig. 1). Considering that growth curves can differ greatly between *P. aeruginosa* strains, we decided to also include a 24-h time point. Environmental and clinical LasR-negative strains behave similarly to the engineered PA14 Δ*lasR* mutant, with production of 3-oxo-C_12_-HSL being augmented upon surface growth when compared to planktonic (Fig. 4). The production profile varies among the LasR-negative backgrounds: strain 18G steadily produces 3-oxo-C_12_-HSL during surface growth. At 24 h, there is sixfold more in surface than in planktonic growth conditions. The environmental strain 32R and the clinical strain E113 have production profiles similar to PA14 Δ*lasR*, and the concentration of 3-oxo-C_12_-HSL peaks at the late exponential phase (Fig. 4; Fig. S3). Production is advanced (compared with PA14 Δ*lasR*) in strains 78RV and E167. In these strains, AHL production peaks at the early exponential phase (Fig. 4; Fig. S3). Finally, upregulation of 3-oxo-C_12_-HSL production upon surface growth was not observed for the clinical strain E41 under our test conditions. Taken together, these results confirm that the absence of a functional LasR generally induces the production of 3-oxo-C_12_-HSL in response to growth in association with surfaces, despite the general requirement of LasR to produce this AHL in standard laboratory planktonic culture conditions.

### 3-oxo-C_12_-HSL induces the expression of pyocyanin in the absence of LasR

**Fig 5:**
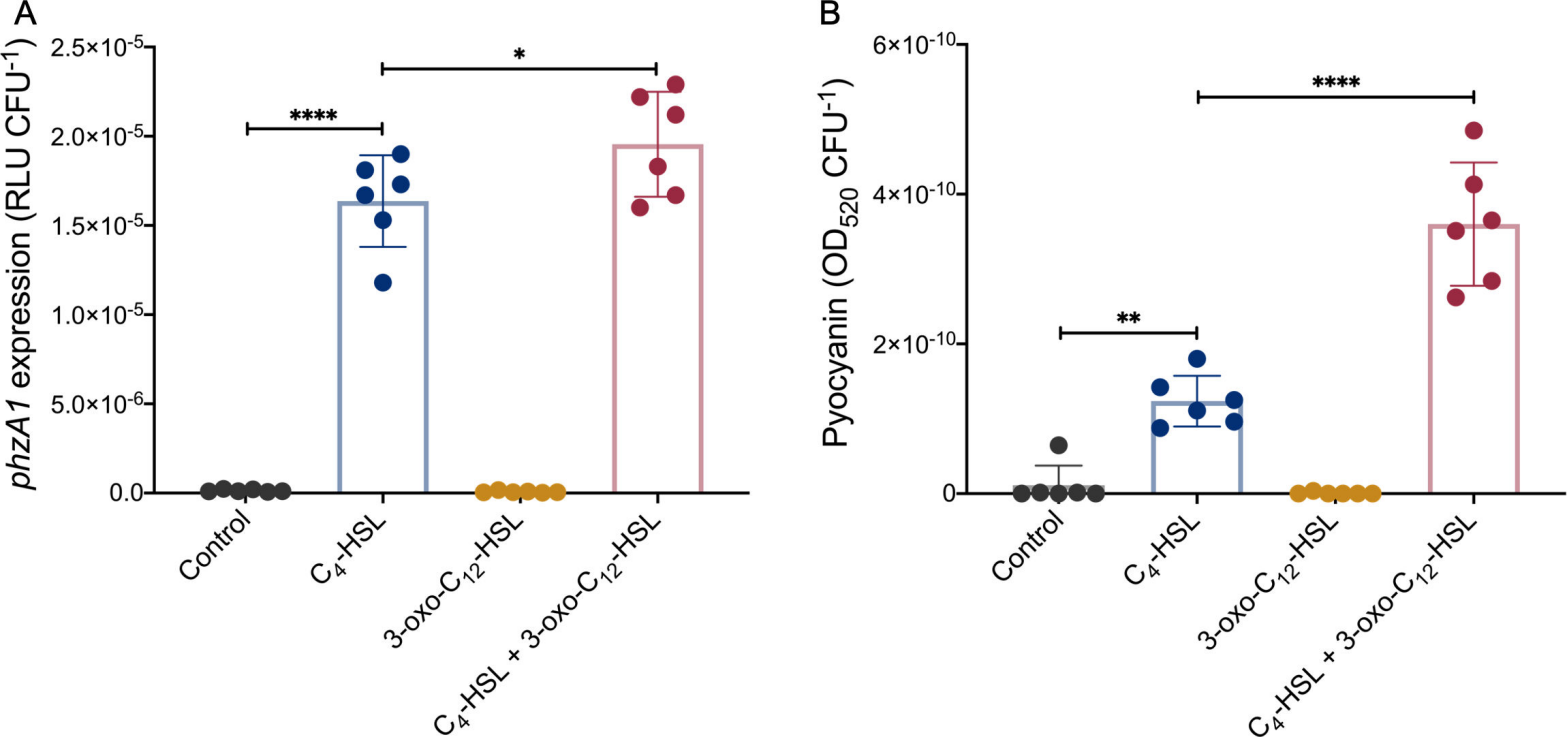
Exogenous 3-oxo-C_12_-HSL induces transcription of the operon *phz1* and pyocyanin production in a *lasR*-negative background. (**A**) Luminescence of the *phzA1-lux* chromosomal reporter was measured in the AHL-negative LasR-defective background (Δ*lasR*Δ*lasI*Δ*rhlI*) after the addition of 1.5 µM of C_4_-HSL, 3 µM of 3-oxo-C_12_-HSL, or both molecules at the late stationary phase (24 h). Acetonitrile alone was used as control. Relative light units were normalized by viable cell counts and shown in RLU CFU^−1^. (**B**) Pyocyanin produced by Δ*lasR*Δ*lasI*Δ*rhlI* in response to exogenous AHLs was chloroform extracted at 24 h. Production was normalized by cell viable counts and shown in OD_520_ CFU^−1^. The values are means ± standard deviations (error bars) from six replicates. Statistical analyses were performed using ANOVA and Tukey’s multiple comparisons posttest with *P ≤ 0.05; ** *P* ≤ 0.01; and ****P ≤ 0.0001.

The conservation of surface-primed induction of 3-oxo-C_12_-HSL production in LasR-defective isolates strongly suggests that this signaling molecule mediates significant biological responses in this context. Because 3-oxo-C_12_-HSL is only/essentially known as the autoinducing ligand of LasR, in a LasR-defective background, its production could be considered as a waste of resources. Thus, a plausible explanation for the conservation is that, in the absence of a functional LasR, 3-oxo-C_12_-HSL remains beneficial when *P. aeruginosa* is growing on a surface. Pyocyanin production relies on the expression of the redundant operons *phzA1B1C1D1E1F1G1* (*phz1*) and *phzA2B2C2D2E2F2G2* (*phz2*)—culminating in the synthesis of phenazine-1-carboxylic acid (PCA). PCA is converted into several phenazines, including pyocyanin, the blue pigment characteristic of *P. aeruginosa* cultures (56). Transcription of the *phz1* operon relies on RhlR (13, 57). To verify if 3-oxo-C12-HSL could be implicated in RhlR-dependent QS, we evaluated the level of transcription from the *phz1* promoter during surface growth, using a chromosomal *phzA1-lux* fusion reporter, in an AHL- and a LasR-negative triple mutant (Δ*lasR*Δ*lasI*Δ*rhlI*). As expected, no transcription is seen in the control condition or when only 3-oxo-C_12_-HSL is provided, and upon addition of exogenous C_4_-HSL, *phz1* transcription is induced, consistent with the requirement of C_4_-HSL for RhlR activity (Fig. 5A). However, unexpectedly, combined addition of C_4_-HSL and 3-oxo-C_12_-HSL further induces the expression of *phz1* (Fig. 5A). The synergetic activation of these signal molecules is also seen for pyocyanin production (Fig. 5B). The concomitant addition of C_4_-HSL and 3-oxo-C_12_-HSL induces by almost threefold the production of this redox-active molecule compared to the addition of C_4_-HSL alone. Similar to the observed *phz1* expression, 3-oxo-C_12_-HSL alone is not sufficient to induce pyocyanin production. These results clearly demonstrate that 3-oxo-C_12_-HSL modulates QS-regulated responses even in the absence of its cognate response regulator LasR. This activity depends on the presence of C _4_ -HSL and thus likely on the function of RhlR.

### 3-oxo-C_12_-HSL produced by LasR-negative strains positively regulates the LasB virulence determinant in cocultures

**Fig 6:**
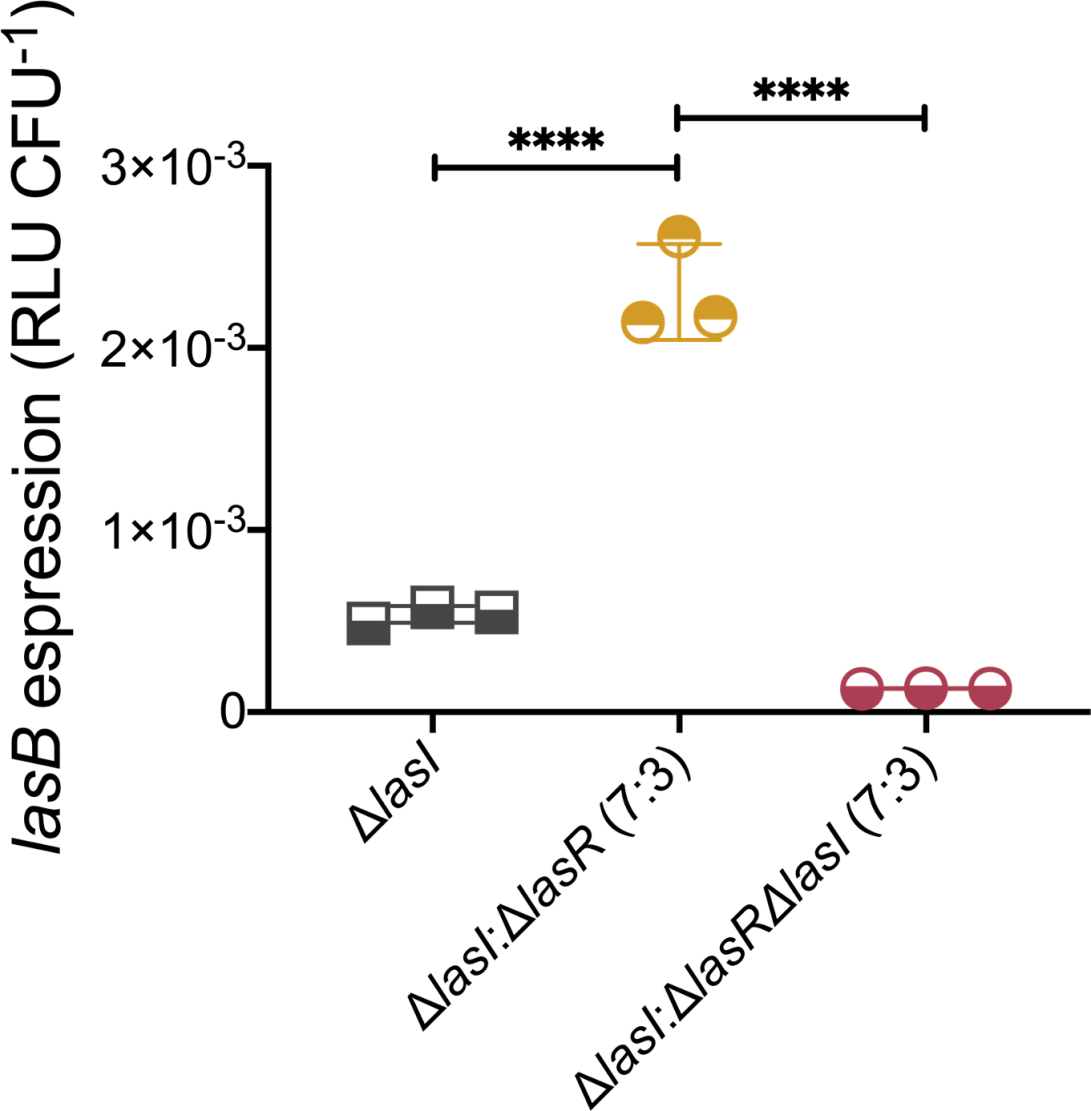
Surface-grown LasR-active cells utilize 3-oxo-C_12_-HSL produced by surrounding LasR-defective mutants, inducing *lasB* expression. Luminescence reading of a *lasB-lux* chromosomal reporter inserted in a LasR-active Δ*lasI* mutant (Δ*lasI*::CTX *lasB-lux*). Monoculture of Δ*lasI* was used as control (gray). Coculture Δ*lasI* and △*lasR* with 7:3 Δ*lasI*-to-Δ*lasR* cell initial ration (yellow). Coculture of Δ*lasI* and Δ*lasR*Δ*lasI* with 7:3 Δ*lasI*-to-Δ*lasR*Δ*lasI* cell initial ratio (red). Relative light unit was normalized by viable cell count of Δ*lasI*::CTX *lasB-lux* strain at 16 h and is shown in RLU CFU^−1^. The values are means ± standard deviations (error bars) from three replicates. Statistical significance was calculated by ANOVA and Tukey’s multiple comparisons posttest with ****P ≤ 0.0001.

AHLs are conserved extracellular intraspecies signaling molecules. Based on this characteristic, we wondered if 3-oxo-C_12_-HSL produced by LasR-defective isolates could be used by surrounding LasR-active cells to induce LasR-dependent factors. These factors include several exoproducts such as proteases (e.g., LasA and LasB) that can be used by the whole population (“public goods”). To verify this, we measured the activity of the chromosomal *lasB-lux* reporter inserted in Δ*lasI* mutant (Δ*lasI*::CTX *lasB-lux* background) in a surface-associated coculture with a Δ*lasR* mutant. Because the Δ*lasI* mutant is unable to produce 3-oxo-C_12_-HSL, the *las* system cannot be activated in this background; however, this strain is LasR-active and prone to induction by exogenous 3-oxo-C_12_-HSL. As expected, *lasB* transcription is at basal levels in Δ*lasI* mutant monoculture (Fig. 6). Coculture with Δ*lasR*, which produces 3-oxo-C_12_-HSL under these surface culture conditions, induces the transcription of the *lasB*-lux reporter by more than fourfold at the late stationary phase in which the concentration of LasR-inducing 3-oxo-C_12_-HSL is at its peak. This upregulation depends solely on the production of 3-oxo-C_12_-HSL by the Δ*lasR* mutant as it is not seen in cocultures with the double-mutant Δ*lasR*Δ*lasI*. Thus, 3-oxo-C_12_-HSL produced by LasR-negative strains can be used by surrounding LasR-active cells, modulating the expression of the QS-regulated genes at the communal level.

### Virulence of *P. aeruginosa* in coinfection settings is partially dependent on the enrichment of 3-oxo-C_12_-HSL provided by LasR-defective cells

**Fig 7:**
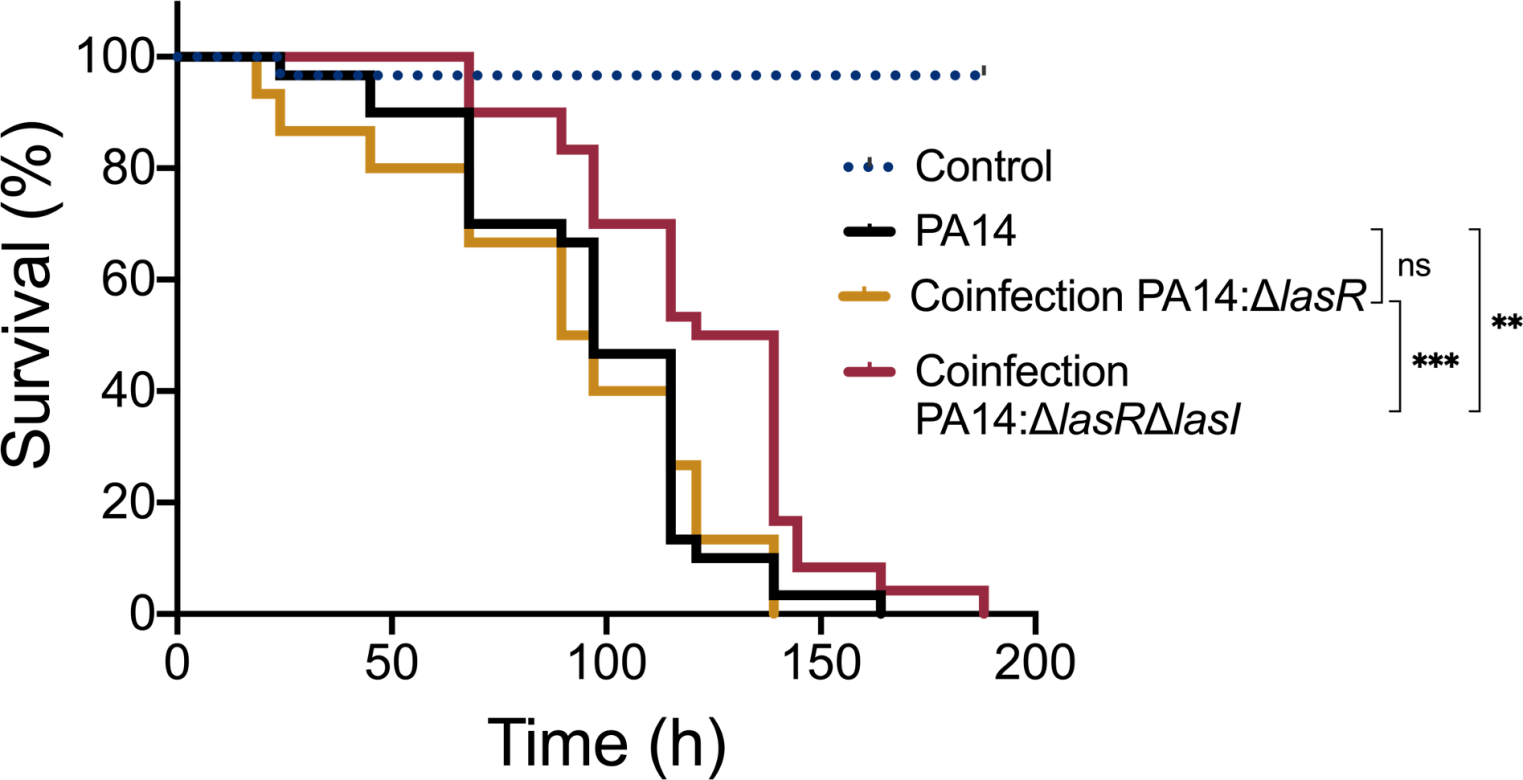
In coinfection settings, full virulence of *P. aeruginosa* toward *D. melanogaster* depends on the provision of 3-oxo-C_12_-HSL produced by Δ*lasR*. Fruit flies were infected with suspended cells in 5% sucrose. Fly survival was monitored over time. *n* = 30 flies per group for each experiment. Experiment was performed independently twice. Statistical significance was determined using Mantel-Cox survival analysis. ns, non-significant, **P ≤ 0.01, and ***P ≤ 0.001.

Even in the absence of a functional LasR or endogenous production of its cognate autoinducer, virulence traits are positively regulated by 3-oxo-C_12_-HSL at individual and community levels (Fig. 5 and 6). Thus, we postulated that a coinfection with a mixture of LasR-responsive and LasR-defective strains would be more virulent than a separate infection with the respective strains. To test this, we used the fruit fly *Drosophila melanogaster* as an infection host—in which *P. aeruginosa* causes a disease and mortality (54). We fed fruit flies with *P. aeruginosa* cells and monitored the survival of the flies for 8 days post-infection. Feeding assay mimics a chronic infection (58). The virulence of WT PA14 (LasR-active) and Δ*lasR* mutant (LasR-defective) was evaluated individually, as well as in a coinfection setting with a 7:3 ratio, respectively (Fig. 7; Fig. S3). Additionally, the virulence of the double mutant Δ*lasR*Δ*lasI* was assessed in both individual and coinfection settings to elucidate the role of 3-oxo-C_12_-HSL in this response (Fig. 7; Fig. S4). Under our conditions, the survival rate of the coinfection with PA14 and Δ*lasR* was comparable to that of the infection with PA14 only throughout the duration of the experiment. In contrast, the coinfection with PA14 and the Δ*lasR*Δ*lasI* double mutant induced less fly mortality. These observations underscore that virulence in coinfection settings with LasR-negative cells is partially dependent on the production of 3-oxo-C_12_-HSL by the latter as it is significantly reduced when this molecule cannot be produced (i.e., double mutant Δ*lasR*Δ*lasI*).

## DISCUSSION

**Fig 8:**
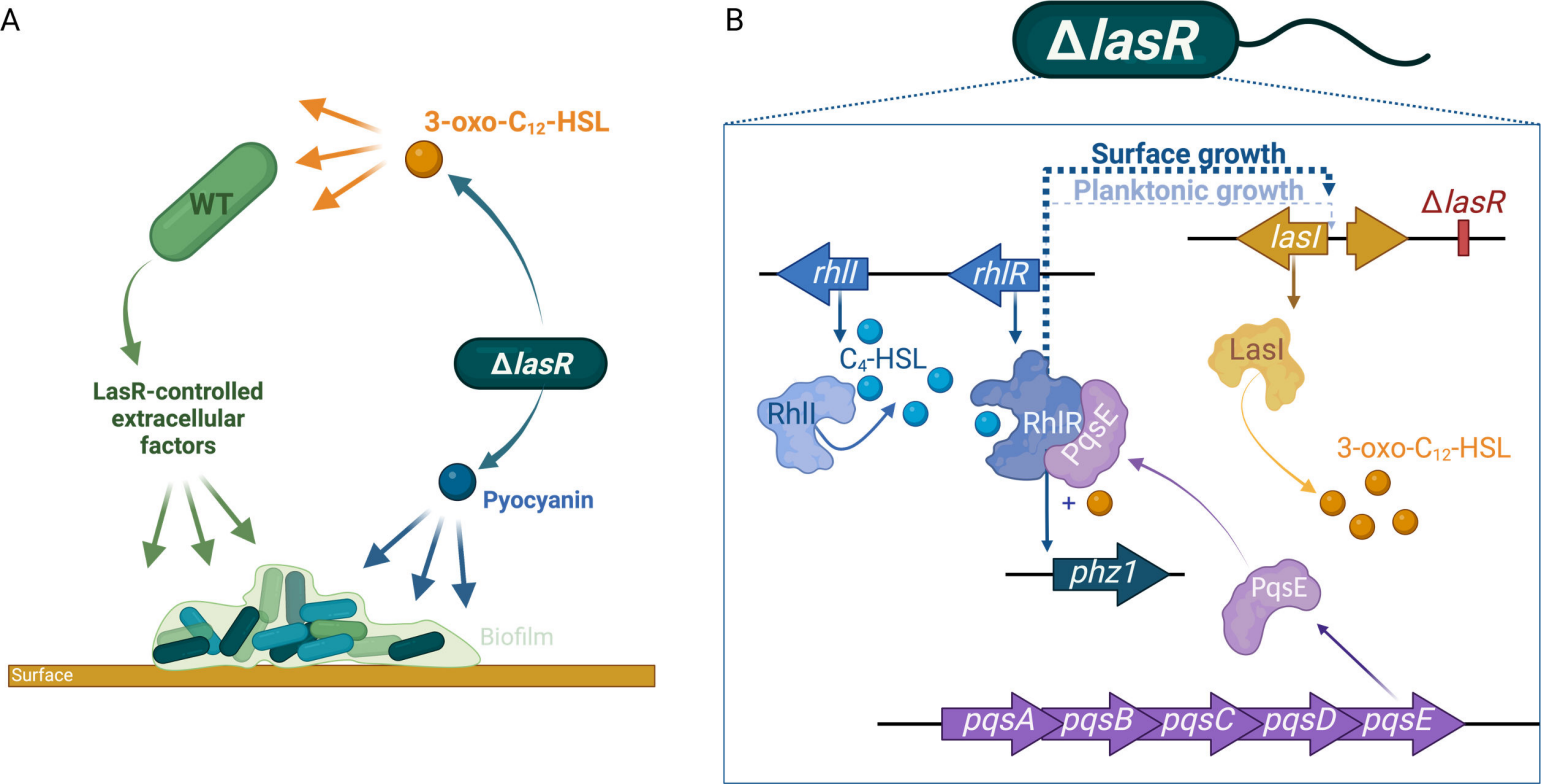
Schematic overview of the investigated QS pathways. (**A**) Interactions in a surface-grown mixed population of *P. aeruginosa*. LasR-negative cells enrich the populational 3-oxo-C_12_-HSL pool, further inducing LasR-controlled extracellular factors and autologous pyocyanin production which benefits the population. (**B**) QS regulation in a LasR-negative cell of *P. aeruginosa* PA14. In the absence of LasR, 3-oxo-C_12_-HSL production is regulated by the active RhlR protein (i.e., in complex with C_4_-HSL and PqsE). This indirect regulation is induced in surface-grown cells (ticker arrow), compared to the planktonic lifestyle (tinner arrow). The presence of 3-oxo-C_12_-HSL appears to modulate the active RhlR complex, inducing the expression of *phz1* and the production of pyocyanin.

The characteristics and behaviors displayed by bacteria within biofilms have been extensively investigated over the years. These surface-associated communities exhibit features that clearly distinguish them from their free-living counterpart. This is due to a sequential and highly regulated process that mediates the transition from planktonic to a sessile lifestyle (59). Although QS regulates social behaviors, often also modulated by aspects related to the sessile way of life, it has been essentially characterized genetically and biochemically in cells grown in broth. In the present study, we show that surface association is sufficient to induce the LasR-independent expression of *lasI* in *P. aeruginosa* and that 3-oxo-C_12_-HSL modulates the expression of virulence determinants even in the absence of the cognate transcriptional regulator LasR (Fig. 8).

Surface sensing has been previously linked to differential bacterial responses. For instance, we have shown that regulation of the small RNAs RsmY/RsmZ is modulated differently in broth versus surface-grown cells, probably aiding bacterial adaptation to growth conditions (60). Similarly, expression of *lasR* increases in a surface-dependent manner, culminating in a surface-primed QS activation, due to the sensitization of surface-grown cells to the cognate AHL 3-oxo-C_12_-HSL (39). Therefore, QS of *P. aeruginosa* responds differently to the same concentration of 3-oxo-C_12_-HSL: weaker QS activation is seen in planktonic cultures, in contrast to high QS activation in surface-associated cells. This mechanism is reported to rely on type IV (TFP) pili retraction as surface-primed *lasR* upregulation is lost in the absence of the motors PilT and PilU (39). Thus, a relationship between QS and surface sensing is established, but its complexity remains to be clearly defined.

LasR-defective *P. aeruginosa* isolates have been generally related to human chronic infections, in which this bacterium persists in the lungs of people with CF as a biofilm. Recently, the generally high occurrence of such isolates challenged this long-held notion (32, 33). Loss of LasR function appears to be a widespread adaptation feature of this bacterium (32). Our results support a model in which surface attachment, a growth condition often encountered by *P. aeruginosa*, induces RhlR-dependent production of 3-oxo-C_12_-HSL in a LasR-defective background—sustaining QS responsiveness in this condition. The mechanism by which RhlR modulates *lasI* expression remains elusive, as in our settings, the transcription of *lasI* was not coupled with the production of 3-oxo-C_12_-HSL. RhlR, as other LuxR-type proteins, is known as a transcriptional regulator, and yet, our results suggest an RhlR-dependent (most likely indirect) posttranscriptional regulation of *lasI*. To our knowledge, the only report of such level of regulation on *lasI* relies on an RNA thermometer and, therefore, cannot explain the regulation observed here (61). The most plausible explanation for the RhlR-dependent production of 3-oxo-C_12_-HSL is that this regulator activates a putative translational regulator required for *lasI* expression; in the absence of RhlR—and consequently, of this RhlR-dependent translational regulator, the translation of *lasI* is blocked and no 3-oxo-C_12_-HSL is produced. And other questions related to this regulation still remain. For instance, why is RhlR-dependent expression of LasI observed in a LasR-deficient background more prominent in sessile cells? Compared to planktonic growth, both sessile LasR-active and LasR-defective cells produce more C_4_-HSL (Fig. S5), which could lead to a stronger activation of the *rhl* system, culminating in the upregulation of RhlR-dependent factors. However, the RhlR-dependent 3-oxo-C_12_-HSL overproduction in sessile cells is seen only in LasR-defective backgrounds (Fig. 8).

Irrespective of the mechanism, the production of 3-oxo-C_12_-HSL appears to have important biological implications. As mentioned before, surface association upregulates LasR, thus sensitizing cells to 3-oxo-C_12_-HSL (39). Upregulation of 3-oxo-C_12_-HSL in a LasR-defective background does not appear dependent on the same mechanism as the TFP retraction motors PilT and PilU are not required for this response (Fig. S6).

The conservation of surface-primed induction of 3-oxo-C_12_-HSL in naturally occurring LasR-defective isolates of *P. aeruginosa* is an indicator of its importance. We observed this response in naturally evolved LasR-defective isolates from both clinical and environmental origins (32, 43). The environmental isolates used here, namely, 18G, 32R, and 78RV, were recently characterized as LasR-defective strains based on their inability to perform LasR-dependent activities in liquid cultures (32). Of note, due to the ability to mediate RhlR-regulated QS, LasR-defective 78RV was characterized as a RAIL strain (32), like the CF isolates E113 and E167, which also have functional RhlR-dependent QS responses (37). In the absence of a functional LasR, surface association induces the production of the 3-oxo-C_12_-HSL signal irrespective of the QS-responsiveness mediated by LasR-independent RhlR. This response is prevalent but not universal. Isolate E41 produces trace concentrations of 3-oxo-C_12_-HSL and its production was not induced by surface-association when compared with planktonic cells. Response variability is not surprising considering the diversity of *P. aeruginosa* isolates, but our results highlight that LasR-deficient *P. aeruginosa* isolated from both clinical and environmental settings are often proficient in the production of 3-oxo-C_12_-HSL when adopting an attached growth mode. Thus, this ability appears to be an intrinsic and beneficial feature of this species.

Mutations in the cognate synthase gene, *lasI*, are much less frequently detected than those found in the *lasR* gene (33). The most accepted explanation for this discrepancy is social cheating. Cheaters are individuals that benefit from a shared beneficial product or function (“public good”) while contributing less than average to the metabolic cost. Inactivation of LasI would not prevent response to 3-oxo-C_12_-HSL produced by neighboring WT cells and thus activate a functional LasR. LasR-defective isolates emerge even in experimental conditions that do not apparently require QS-induced products (and thus cheating) (62). An alternative explanation for a lower frequency of *lasI*-null isolates is that 3-oxo-C_12_-HSL might contribute an alternative function beyond LasR activation. This interpretation is supported by our results, where LasR-defective strains retain the ability to respond to the presence of 3-oxo-C_12_-HSL. Indeed, the expression of *phz1*, a QS-regulated operon required for pyocyanin production, is controlled by RhlR and its cognate ligand C_4_-HSL. Concomitant addition of 3-oxo-C_12_-HSL further induces *phz1* transcription and positively regulates pyocyanin production suggesting a response to this non-cognate AHL (Fig. 5). The induction of RhlR-controlled *phz1* expression by 3-oxo-C_12_-HSL is also seen in the double mutant Δ*lasR*Δ*lasI* (Fig. S7). Basal expression of *phz1* is due to the self-produced C_4_-HSL. The addition of 3-oxo-C_12_-HSL further enhances *phz1* transcription activity, and the highest expression is seen when C_4_-HSL is added with 3-oxo-C_12_-HSL. The requirement of C_4_-HSL to induce the transcription of *phz1* by 3-oxo-C_12_-HSL indicates that this response is RhlR dependent, as proposed in Fig. 8. However, it is possible that other regulatory factors also contribute to this regulation. *P. aeruginosa* possesses a third LuxR-type regulator named QscR (63). Unlike LasR and RhlR, QscR does not have a cognate AHL synthase (63). Interestingly, QscR is a promiscuous receptor capable of binding to various long-chain AHLs, including 3-oxo-C_12_-HSL (64, 65). In a wild-type background, QscR suppresses pyocyanin production (63). However, in the absence of LasR, the dynamics of QS regulation are reconfigured, and the contribution of QscR to pyocyanin production is conceivable. Alternatively, 3-oxo-C_12_-HSL could partially induce a LuxR-independent response. Indeed, such regulation has been described in *P. aeruginosa* (66). The addition of exogenous AHLs, both self- and non-self-produced, elicited a response in a LuxR-null background (i.e., in the absence of LasR, RhlR, and QscR). However, LuxR-independent responses were not found to modulate the expression of genes associated with pyocyanin production (66). This observation reduces the likelihood of 3-oxo-C_12_-HSL inducing pyocyanin production through this particular regulatory pathway.

Producing 3-oxo-C_12_-HSL in the absence of LasR can also have a positive community outcome. Because it is exported, we have shown that this AHL can have exogenous effects in surrounding cells in a surface-associated setting (Fig. 8). Thus, localized production of 3-oxo-C_12_-HSL by LasR-negative clusters could induce the expression of QS-regulated virulence factors in LasR-active cells, with minimal metabolic cost to the LasR-negative producers. Moreover, the production profile is delayed in LasR-defective strains when compared to the WT. Therefore, the mixed population composed of both LasR-active and LasR-defective cells would be subjected to steady levels of 3-oxo-C_12_-HSL.

Furthermore, in natural habitats, *P. aeruginosa* is typically part of complex polymicrobial communities. Microbes within these communities can actively respond to one another, and these interactions range from cooperation to competition (67). For example, in mixed populations consisting of wild-type and LasR-negative cells, QS-controlled molecules are positively regulated. This regulation relies on reciprocal cross-feeding between the populations. The release of the siderophore pyochelin by LasR-negative cells induces the production of citrate by the wild-type counterpart. In turn, citrate positively regulates RhlR activity in LasR-negative cells, leading to the induction of QS responses (53). Similarly, the continuous production of 3-oxo-C_12_-HSL by *P. aeruginosa* may play a significant role in shaping the biological activities of the population. These examples emphasize the importance of the exchange of molecules within mixed populations, which modulates population dynamics. Furthermore, apart from influencing dynamics within *P. aeruginosa* populations, 3-oxo-C_12_-HSL may also impact interspecies communications.

In this context, LuxR homologs BtaR1 and BtaR2, from *Burkholderia thailandensis*, are promiscuous and can be activated by 3-oxo-C_12_-HSL (68). It is worth noting that both *P. aeruginosa* and *B. thailandensis* are soil saprophytes that can inhabit similar environmental niches. The ecological importance of perceiving signals produced by neighboring cells becomes apparent with the presence of an orphan LuxR homolog (SdiA) in *Salmonella enterica* serovar Typhimurium, a bacterium unable to produce AHLs (69). This ability to “eavesdrop” on AHL signals produced by other bacteria is likely not exclusive to this bacterium and may influence interactions between different species. It provides a rationale for the sustained production of 3-oxo-C_12_-HSL in *P. aeruginosa*. Further support for this idea comes from *Ruegeria* sp., a bacterium associated with marine sponges that possesses a solo LuxI homolog and cannot employ this molecule in a conventional QS-regulated pathway (70).

QS signals also play a pivotal role in host-pathogen interactions. QS-regulated molecules can act as interkingdom QS signals, thus responsible for the communication of bacteria with mammalian cells and the modulation of host immune systems. Indeed, this was reported for 3-oxo-C_12_-HSL [recently reviewed in reference (71)]. Due to the long acyl chain of this autoinducer, the molecule has lipophilic properties and, by directly interacting with biological membranes, can enter mammalian cells and interact with intracellular molecules (72). The presence of 3-oxo-C_12_-HSL induces apoptosis of hematopoietic cells and cytotoxicity of non-hematopoietic cells, including those of the airway epithelium (73 – 77). The host immune responses are also suppressed by 3-oxo-C_12_-HSL, negatively impacting cytokines production, T-cell differentiation, and the function of antigen-presenting cells (78 – 80). Thus, this signal molecule is central to the virulence and pathogenesis of *P. aeruginosa,* and the sustained production of 3-oxo-C_12_-HSL by biofilm-growing LasR-deficient isolates in infected hosts might account for worse clinical outcomes. In infected hosts, could the immunomodulatory activity of 3-oxo-C_12_-HSL, rather than its role as quorum sensing signal, justify the regulatory by-pass in the absence of LasR?

Sustained production of 3-oxo-C_12_-HSL in the absence of LasR in response to surface growth, the most common lifestyle adopted by *P. aeruginosa* in its natural environments*,* appears to be beneficial to the colonization of many environmental niches. Combined with the widespread feature underlying the emergence of LasR-defective isolates, it raises an important question: do these isolates emerge solely to benefit from the cooperating individuals or could they play a positive role in shaping the bacterial community responses?

## SUPPLEMENTAL MATERIAL

Supplemental figures mbio.00922-23-s0001.pdf Fig. S1 to S7.

ASM does not own the copyrights to Supplemental Material that may be linked to, or accessed through, an article. The authors have granted ASM a non-exclusive, world-wide license to publish the Supplemental Material files. Please contact the corresponding author directly for reuse.
