# Supplementary material for: Surface growth of Pseudomonas aeruginosa reveals a regulatory effect of 3-oxo-C12-homoserine lactone in the absence of its cognate receptor, LasR
Source: mBio. 2023 Sep 21;14(5):e00922-23. doi: 10.1128/mbio.00922-23 (PMC10653899; doi:10.1128/mbio.00922-23)
Supplement: Supplemental figures — Fig. S1 to S7. [file mbio.00922-23-s0001.pdf]

Surface growth of *Pseudomonas aeruginosa* reveals a regulatory effect of 3-oxo-C_{12}-homoserine lactone in absence of its cognate receptor, LasR.

Thays de Oliveira Pereira[1], Marie-Christine Groleau[1], Eric Déziel[1#]

[1] Centre Armand-Frappier Santé Biotechnologie, Institut National de la Recherche Scientifique (INRS), Laval, Québec, H7V 1B7, Canada

SUPPLEMENTAL MATERIAL

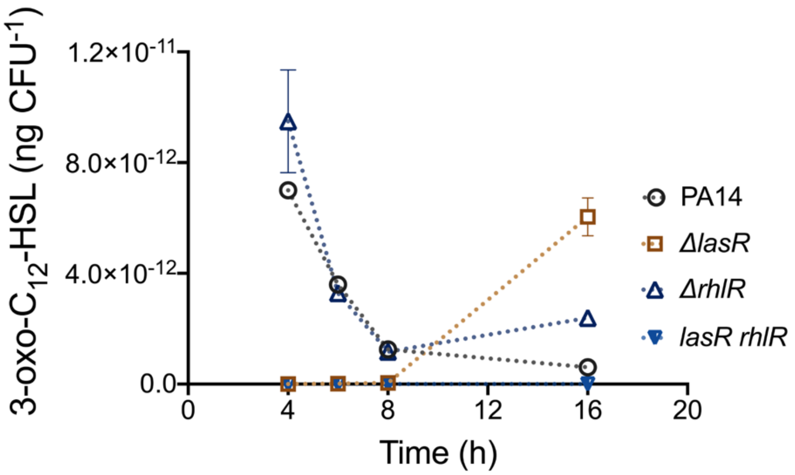

Figure S1. Production of 3-oxo-C_{12}-HSL does require RhlR in LasR-inactive cells. Concentration of 3-oxo-C12-HSL was measured at different time points during surface growth by LC/MS. Values were normalized by the viable cell counts and shown in ng CFU^{-1}. Values are means ± standard deviation (error bars) from three replicates.

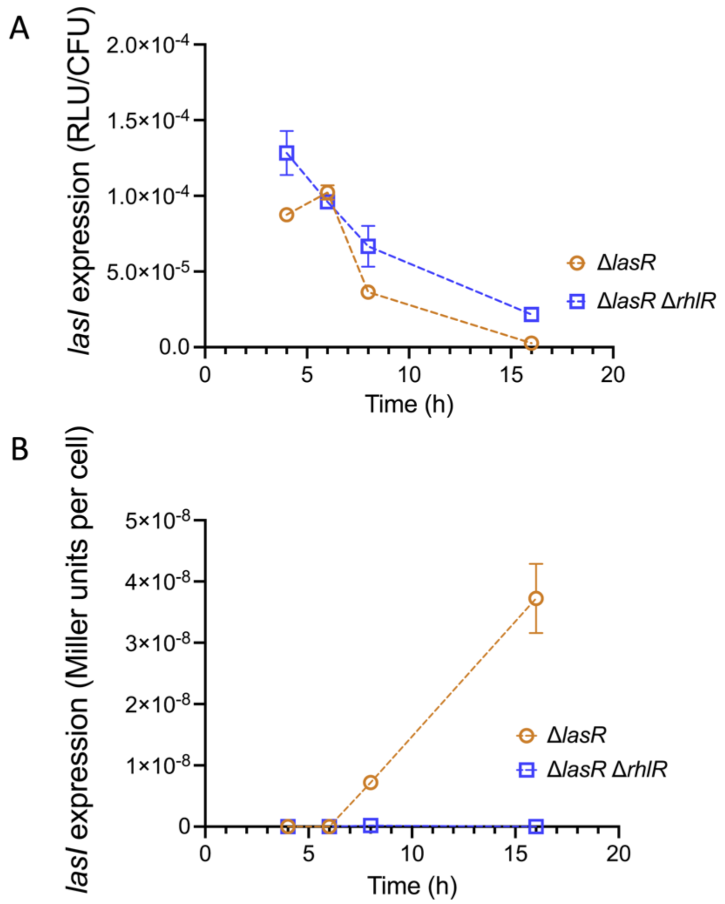

Figure S2. RhlR controls the translational expression of *lasI* in surface-grown cells. (A) Transcription activation from the chromosomal *lasI-lux* reporter in the backgrounds Δ*lasR* and Δ*lasR*Δ*rhlR* at different time points during surface growth. **(B)** The translational activity of *lasI* was measured in the same conditions via β-galactosidase activity of a *lasI′–′lacZ* translational fusion (pME3853). The values are means ± standard deviation (error bars) from three replicates.

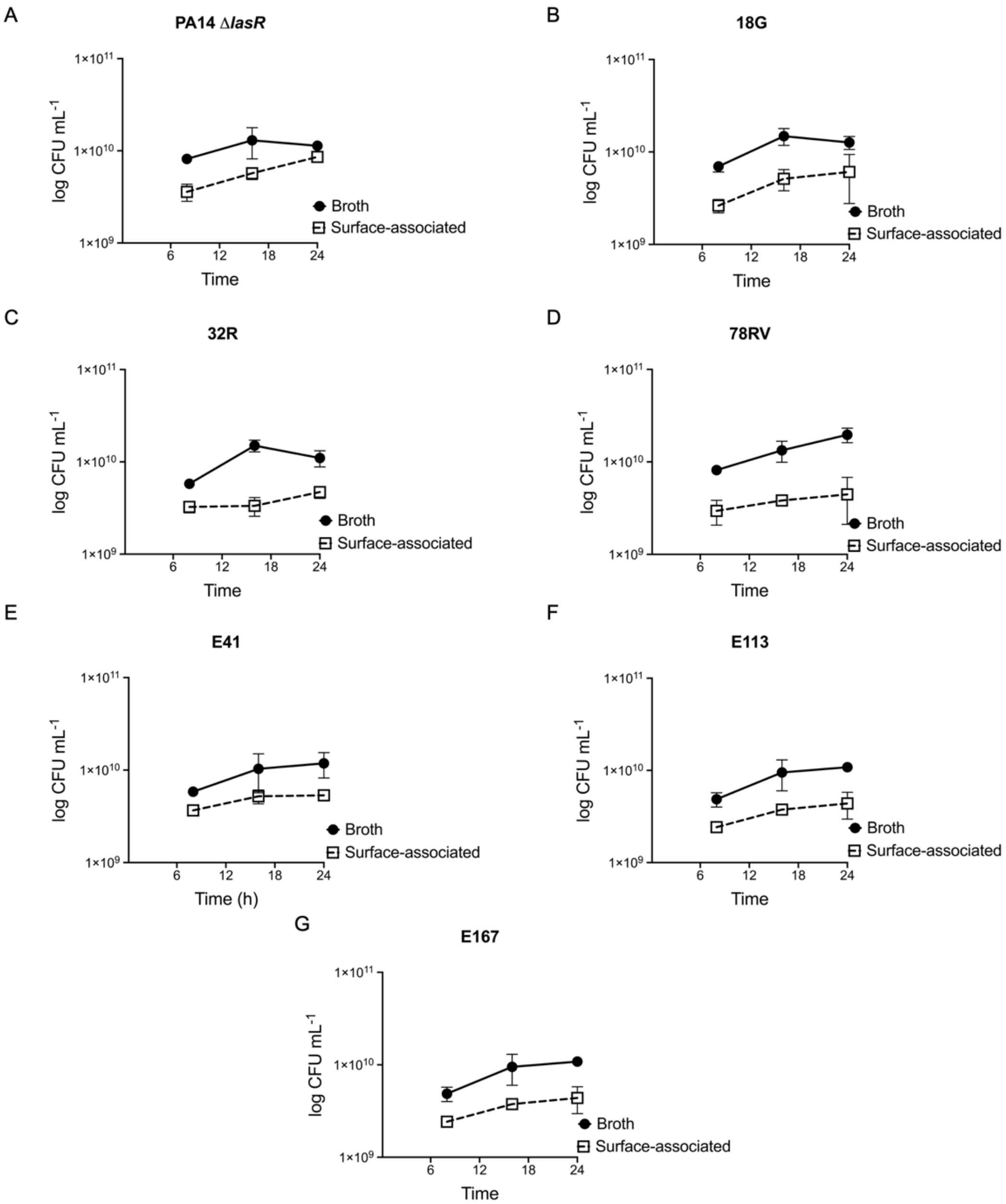

Figure S3. Growth profile of natural occurring LasR-defective isolates. Growth in broth and surface conditions was determined by the count of viable cells per millilitre (CFU mL^{-1}). This data is complementary to the one shown in Figure 4. The values are means ± standard deviation (error bars) from three replicates.

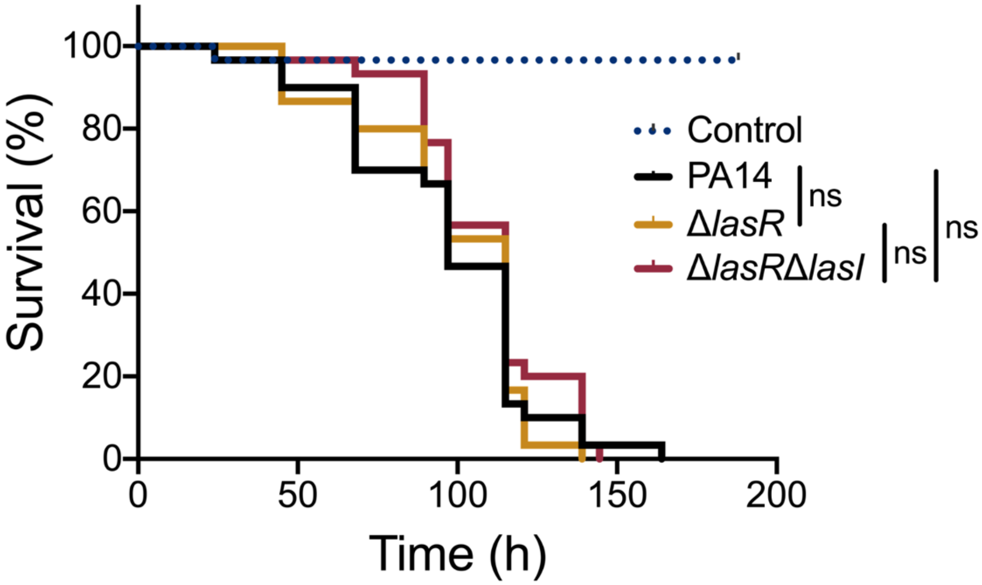

Figure S4. Functionality of the *las* system is not required for *P. aeruginosa* virulence toward *D. melanogaster*. Fruit flies were infected with suspended cells in 5% sucrose. Fly survival was monitored over time. *n* = 30 flies per group for each experiment. Experiment was performed independently twice. Statistical significance was determined using the Kaplan-Meier survival analysis. ns, non-significant.

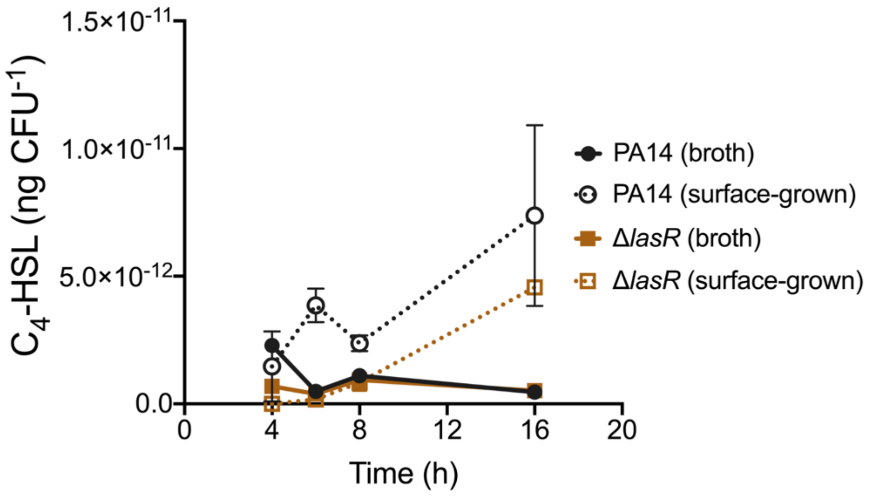

Figure S5. Surface growth induces C_4-HSL production in both PA14 and its isogenic *lasR* mutant. C_4-HSL concentration was measured in PA14 and the isogenic *lasR* mutant at different time points during planktonic (broth culture) and surface growth (surface of agar-solidified culture media) by LC/MS. Values were normalized by the viable cell counts and shown in ng CFU^{-1}. The values are means ± standard deviation (error bars) from three replicates.

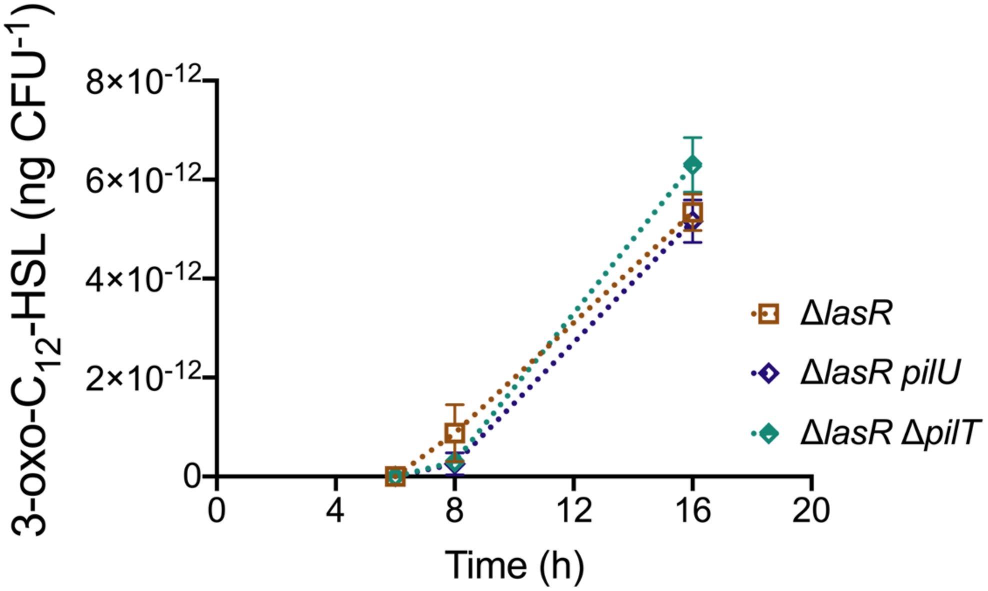

Figure S6. Type IV pili motors PilU and PilT are not responsible for surface-primed 3-oxo-C_{12}-HSL induction. 3-oxo-C_{12}-HSL concentration was measured in PA14 Δ*lasR* and the double mutants Δ*lasR pilU* and Δ*lasR* Δ*pilT* at different time points during surface growth by LC/MS. Values were normalized by the viable cell counts and shown in ng CFU^{-1}. The values are means ± standard deviation (error bars) from three replicates.

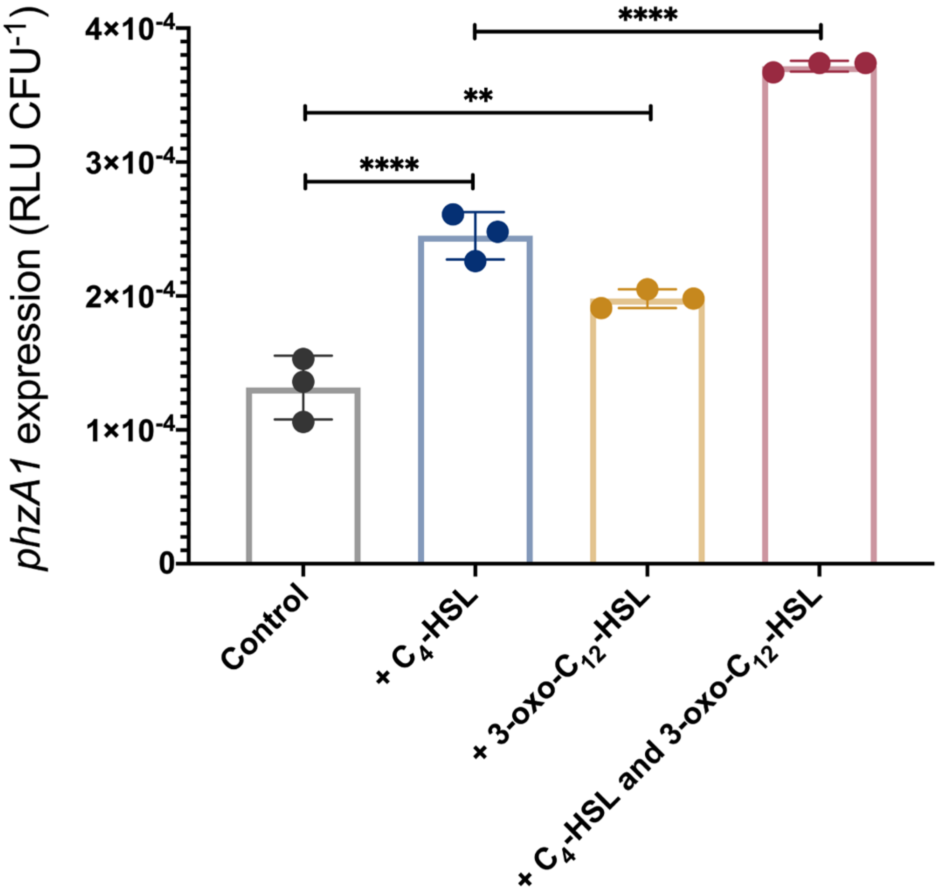

Figure S7. Induction of the *phz1* operon by the 3-oxo-C_{12}-HSL is also seen with endogenous C_4-HSL. Luminescence of the *phzA1-lux* chromosomal reporter was measured in a *las* system negative background (Δ*lasR*Δ*lasI*) after the addition of 1.5 μM of C_4-HSL, 3 μM of 3-oxo-C_{12}-HSL or both molecules at 8h. Solvent alone was used as control. Relative light units were normalized by by viable cell count and is shown in RLU CFU^{-1}. Statistical analyses were performed using one-way analysis of variance (ANOVA) and Tukey's multiple comparisons posttest with ** $P \leq 0.01$ and **** $P \leq 0.0001$.
